# The prognostic effect of ethnicity for gastric and esophageal cancer: the population-based experience in British Columbia, Canada

**DOI:** 10.1186/1471-2407-11-164

**Published:** 2011-05-09

**Authors:** Morteza Bashash, T Greg Hislop, Amil M Shah, Nhu Le, Angela Brooks-Wilson, Chris D Bajdik

**Affiliations:** 1 Cancer Control Research Program, BC Cancer Agency, Vancouver, Canada; 2 Interdisciplinary Oncology Program, University of British Columbia, Vancouver, Canada; 3 Medical Oncology, BC Cancer Agency, Vancouver, Canada; 4 Canada's Michael Smith Genome Sciences Centre, BC Cancer Agency, Vancouver, Canada; 5 Department of Medicine, University of British Columbia, Vancouver, Canada; 6 School of Population and Public Health, University of British Columbia, Vancouver, Canada; 7 Department of Statistics, University of British Columbia, Vancouver, Canada; 8 Department of Biomedical Physiology and Kinesiology, Simon Fraser University, Vancouver, Canada

## Abstract

**Background:**

Gastric and esophageal cancers are among the most lethal human malignancies. Their epidemiology is geographically diverse. This study compares the survival of gastric and esophageal cancer patients among several ethnic groups including Chinese, South Asians, Iranians and Others in British Columbia (BC), Canada.

**Methods:**

Data were obtained from the population-based BC Cancer Registry for patients diagnosed with invasive esophageal and gastric cancer between 1984 and 2006. The ethnicity of patients was estimated according to their names and categorized as Chinese, South Asian, Iranian or Other. Cox proportional hazards regression analysis was used to estimate the effect of ethnicity adjusted for patient sex and age, disease histology, tumor location, disease stage and treatment.

**Results:**

The survival of gastric cancer patients was significantly different among ethnic groups. Chinese patients showed better survival compared to others in univariate and multivariate analysis. The survival of esophageal cancer patients was significantly different among ethnic groups when the data was analyzed by a univariate test (p = 0.029), but not in the Cox multivariate model adjusted for other patient and prognostic factors.

**Conclusions:**

Ethnicity may represent underlying genetic factors. Such factors could influence host-tumor interactions by altering the tumor's etiology and therefore its chance of spreading. Alternatively, genetic factors may determine response to treatments. Finally, ethnicity may represent non-genetic factors that affect survival. Differences in survival by ethnicity support the importance of ethnicity as a prognostic factor, and may provide clues for the future identification of genetic or lifestyle factors that underlie these observations.

## Background

Gastric and esophageal cancers are among the most lethal human malignancies. Worldwide, gastric cancer is the fourth most common cancer, but the second most common cause of death from cancer [1]. Esophageal cancer is the eighth most common cancer, but the sixth most common cause of cancer death [1]. The epidemiology of these cancers is geographically diverse. Incidence rates for gastric cancer vary from 3.4 per 100,000 among women in North America to 26.9 per 100,000 among men in Asia. The 5-year survival is usually about 20% [2]; however, countries with higher incidence rates of gastric cancer generally have better survival rates than countries with lower incidence [3]. Incidence rates for esophageal cancer range from 5-10 per 100,000 in North America to more than 100 per 100,000 in Eastern Iran near the Caspian Sea [4]. The differences between populations reflect environmental and lifestyle (including healthcare) factors, as well as genetic profiles [5].

In order to investigate risk associated with genetic characteristics of a population (*ie*. ethnicity) and to reduce or eliminate environmental confounding, it is preferable to conduct a study in a single geographic area with a heterogenous population rather than to conduct international comparisons [5]. British Columbia (BC), Canada, has a multi-ethnic population. Based on 2006 census data, about one in every four of the 4,428,400 British Columbians (24.8%) belongs to a visible minority, representing about one million people in the province. Visible minority is a category that includes persons who are non-Caucasian in race or non-white in colour and who do not report being Aboriginal http://www.statcan.gc.ca/concepts/definitions/minority01-minorite01a-eng.htm. Of these, approximately 75% were born outside Canada, and about 60% immigrated to BC from 1991 to 2006 [6]. That indicates about 676,000 immigrants and 297,000 non-immigrants in BC belonged to a visible minority group in 2006 [6]. Chinese was the largest group, accounting for 40% of all visible minorities in the province, followed by South Asians (26%) [6]. Iranians represent a relatively small but growing percentage of the BC population (0.5%, or 19,000 people) in 2001 [7], although they originate from a geographic region with the world's highest incidence of gastric and esophageal cancers [8,9]. This study compares survival of gastric and esophageal cancer patients among Chinese, South Asian and Iranian and other ethnic groups in BC.

## Methods

This study received approval from the Research Ethics Board at the BC Cancer Agency (BCCA). The study uses historical patient records and, accordingly, patients were not re-contacted. Cancer incidence and survival data for invasive primary esophageal and gastric cancers were obtained from the population-based BC Cancer Registry (BCCR) for all BC patients diagnosed between 1984 and 2006. The BCCR receives national information regarding the vital status of patients and is updated accordingly. The topology and histology of cases were coded according to the International Classification of Diseases for Oncology, Third Edition (ICD-O) [10] for greater coherence with registry information recorded during the entire study time period. The topography for esophageal cancers was then grouped into four categories: esophagus upper third (ICD-O codes C15.0-C15.3), esophagus middle third (ICD-O codes C15.4), esophagus lower third and overlapping lesions (ICD-O codes C15.5), and esophagus unknown (ICD-O codes C15.8 and C15.9). The topography for gastric cancer was grouped into three categories: proximal third (cardia) in the gastroesophageal junction or upper third of the stomach (ICD-O codes C16.0 and C16.1), distal stomach or lower two thirds of the stomach (ICD-O codes C16.2-C16.7), and unknown or unspecified/overlapping lesion (ICD-O codes C16.8 and C16.9). Histological categories for esophageal cancers were squamous cell carcinoma (ICD-O codes 8050-8082), adenocarcinoma (ICD-O codes 8140-8573) and others (mainly ICD-O codes 8000-8020). Histology for gastric cancer was also categorized based on the Lauren classification system as diffuse or intestinal type [11] (diffuse gastric tumors defined by histology codes 8142, 8145 and 8490) [12]. For both esophageal and gastric cancers, nonepithelial tumors (ICD-O codes 8800-9759) were excluded.

Primary treatment was categorized as surgery, chemotherapy and radiotherapy, with only therapeutic (i.e., not diagnostic) surgeries being considered as treatment. Some patients received more than one type of primary treatment, but other information, including information about adjuvant therapy and individual hospitals attended, was not available. Overall survival was the primary study outcome, and was calculated as the time between diagnosis and death. Complete follow-up information was available for all patients to 31 August 2007.

The ethnicity of patients was determined according to their names and categorized as Chinese, South Asian or Iranian. This method for identification of ethnicity was necessary because the BCCR does not record ethnicity or place of birth. Two sources were used to generate surname listings for each of the three ethnic groups: local telephone directories and the Screening Mammography Program of BC (SMPBC; a population-based screening program serving nearly 50% of the age-eligible female population in BC) database. The names in local telephone directories were reviewed manually to identify Chinese, South Asian and Iranian surnames; this was done by several members of the research team from each of the respective ethnic groups. In addition, since the SMPBC database retains both 'place of birth' and 'ethnic group' as reported by the client, all surnames were listed from this source for Chinese women reporting 'Chinese' as their ethnicity, South Asian women reporting 'India', and Iranian women reporting 'Iran'. The same members of the research team reviewed these surname listings and eliminated names that were not typically Chinese, South Asian or Iranian, or which were common to other population groups. This method to identify ethnicity has been used in a number of other studies [7,13-16]] and the methodology has been discussed elsewhere [17-19]. Patients not classified as belonging to any of these three ethnic groups were categorized as "Other." Based on the ethnic distribution of the BC population, more than 80% of "Other" are British and Western Europeans [20]. British and Western Europeans could not be separated as a group because corresponding name lists do not exist.

Univariate comparisons of demographic, tumor and treatment variables between ethnic groups were performed using Chi-square tests. Survival was calculated using the Kaplan-Meier method and log-rank tests were used to compare survival differences among groups. All analyses were performed separately for non-metastatic (Stage I-III) and metastatic (Stage IV) disease. Cox proportional hazards regression was used to estimate the effect of ethnicity adjusted for patient sex, age (less than 55 years, 55-64 years, 65-74 years and 75+ years), date of diagnosis (1984-1990, 1991-1995, 1996-2000, 2001-2006), tumor histology (intestinal and diffuse for gastric cancer; adenocarcinoma and squamous cell carcinoma for esophageal cancer), tumor location, disease stage and primary treatment received (surgery, radiotherapy and/or chemotherapy). The hazard ratio (HR) was calculated for each ethnic group and is the ratio of the hazard rate in each ethnic group compared to the "Other" group. For each HR, a 95% confidence interval (95%CI) was calculated. p-values less than 0.05 were considered statistically significant.

## Results

### Gastric cancer

**Figure 1:**
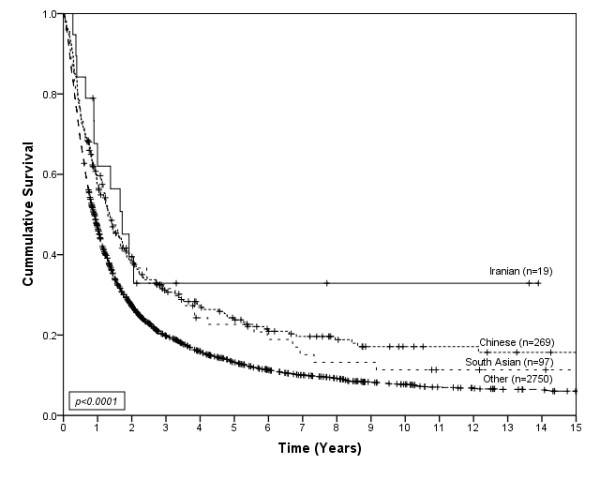
**Survival of gastric cancer patients by ethnic group**.

**Figure 2:**
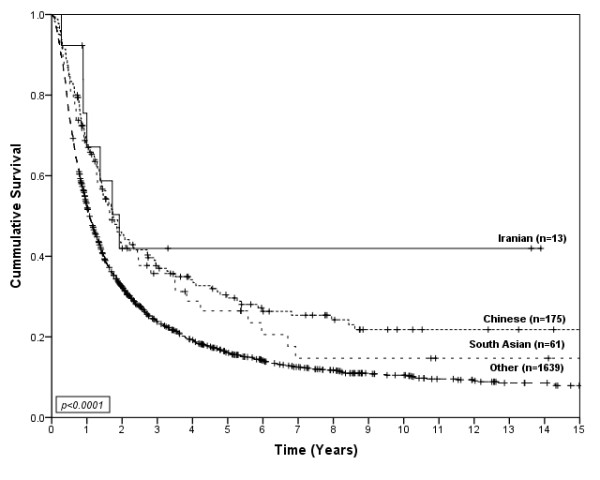
**Survival of gastric cancer patients by ethnic group for non-metastatic disease**.

**Figure 3:**
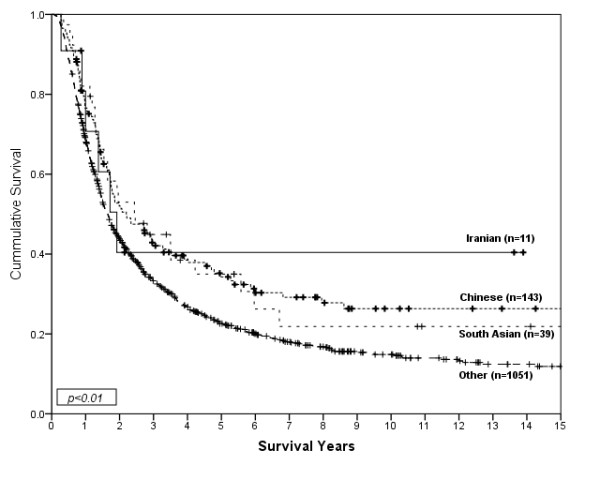
**Survival of gastric cancer patients who received surgery by ethnic group for non-metastatic disease**.

3136 cases of invasive gastric cancer were diagnosed during the study period. Descriptive information for the cases is shown by ethnicity in Table 1. The age and sex distributions were significantly different among the ethnic groups (p < 0.01). A higher proportion of Chinese and South Asian gastric cancer patients were female as compared to the other ethnic groups. The average age at diagnosis was 61.0 years for Iranians, 62.6 years for Chinese, 61.7 years for South Asians, and 65.4 years for Other ethnicities. There were significant differences among the year of diagnosis by ethnicity (p < 0.01). Tumor location was significantly different among the ethnic groups (p < 0.01). Tumors in the proximal 1/3 of the stomach were more common in South Asians and Other ethnicities as compared to Chinese and Iranians. Histology based on the Lauren classification was also significantly different among ethnic groups (p = 0.03). The diffuse type of gastric cancer was most common among Chinese compared to the other ethnic groups. The distribution of stage and proportion with metastatic disease was not significantly different among the ethnic groups; however, treatment by surgery and chemotherapy were significantly different among the ethnic groups. The Chinese and Iranian groups received surgery more often than people in the South Asian or Other groups (p < 0.01), and the South Asian and Iranian groups received chemotherapy more often than Chinese or Others (p < 0.01). Among treated groups; 61% of Iranians, 47% of Chinese, 54% of South Asians and 41% of Others received more than one type of primary treatment (*ie*., Surgery+Chemotherapy+Radiotherapy, Surgery+Chemotherapy, Surgery+Radiotherapy or Chemotherapy+Radiotherapy). Each type of primary treatment was included as a separate variable in multivariate models. Iranians had a median survival of 20 months (95%CI; 10.6-29.4), Chinese had a median survival of 16 months (95%CI; 12.5-19.1), South Asians had a median survival of 15 months (95%CI; 11.2-18.1) and Others had a median survival of 10 months (95%CI; 9.4-10.7). Figure 1 shows survival curves for gastric cancer patients according to ethnic group. Survival was significantly different between ethnic groups (p < 0.01). Iranians (HR = 0.62, 95%CI; 0.31-0.96), South Asians (HR = 0.87, 95%CI; 0.59-0.94) and Chinese (HR = 0.77, 95%CI; 0.61-0.81) showed better survival than people in the Other category. When considered separately by presence or absence of metastatic disease, statistically significant differences were only found for non-metastatic disease (p < 0.01), as shown in Figure 2. South Asians (HR = 0.72, 95%CI; 0.54-0.97) and Chinese (HR = 0.64, 95%CI; 0.53-0.76) showed better survival than the Other category. The survival of Iranians (HR = 0.50, 95%CI; 0.24-1.04) was also better than people in the Other category, but the small number of Iranians (and wide confidence interval) does not exclude the possibility that this is due to chance. Furthermore, the association between survival and ethnicity was only significant for patients with non-metastatic disease who received therapeutic surgery (p < 0.01), as shown in Figure 3.

**Table 1:** Descriptive characteristics for gastric cancer by ethnicity

		Iranian	Chinese	South Asian	Other	p
**Sex (N = 3136)**	*Male*	15 (78.9%)	168 (62.2%)	57 (58.8%)	1974 (71.8%)	*p < 0.001*
		
	*Female*	4 (21.1%)	102 (37.8%)	40 (41.2%)	776 (28.2%)	

**Age in years (N = 3136)**	*Less than 55*	7 (36.8%)	86 (31.9%)	26 (26.8%)	515 (18.7%)	*p < 0.001*
		
	*55-64*	3 (15.8%)	49 (18.1%)	20 (20.6%)	652 (23.7%)	
		
	*65-74*	7 (36.8%)	65 (24.1%)	35 (36.1%)	884 (32.1%)	
		
	*75 and More*	2 (10.5%)	70 (25.9%)	16 (16.5%)	699 (25.4%)	

**Years of Diagnosis (N = 3136)**	*1984-1990*	0 (0.0%)	32 (11.9%)	11 (11.3%)	643 (23.4%)	*p < 0.001*
		
	*1991-1995*	7 (36.8%)	54 (20.0%)	16 (16.5%)	481 (17.5%)	
		
	*1996-2000*	4 (21.1%)	63 (23.3%)	27 (27.8%)	626 (22.8%)	
		
	*2001-2006*	8 (42.1%)	121 (44.8%)	43 (44.3%)	1000 (36.4%)	

**Tumor Histology - Lauren classification (N = 3136)**	*Intestinal*	14 (73.7%)	205 (75.9%)	74 (76.3%)	2188 (79.6%)	*0.032*
		
	*Diffuse*	3 (15.8%)	55 (20.4%)	13 (13.4%)	382 (13.9%)	
		
	*Other*	2 (10.5%)	10 (3.7%)	10 (10.3%)	180 (6.5%)	

**Tumor Location (N = 3136)**	*Proximal 1/3*	6 (31.6%)	52 (19.3%)	47 (48.5%)	1302 (47.3%)	*p < 0.001*
		
	*Distal 2/3*	10 (52.6%)	171 (63.3%)	28 (28.9%)	894 (32.5%)	
		
	NES/NOS*	3 (15.8%)	47 (17.4%)	22 (22.7%)	554 (20.1%)	

**Tumor Stage (N = 2567)**	*I*	1 (5.6%)	14 (6.1%)	3 (3.7%)	108 (4.8%)	0.85
		
	*II*	6 (33.3%)	65 (28.5%)	29 (35.8%)	702 (31.3%)	
		
	*III*	6 (33.3%)	96 (42.1%)	29 (35.8%)	829 (37.0%)	
		
	*IV*	5 (27.8%)	53 (23.2%)	20 (24.7%)	601 (26.8%)	

**Surgery (N = 3080)**	*Yes*	14 (73.7%)	178 (66.7%)	56 (57.7%)	1502 (55.7%)	*0.0027*
		
	*No*	5 (26.3%)	89 (33.3%)	41 (42.3%)	1195 (44.3%)	

**Chemotherapy (N = 3065)**	*Yes*	10 (52.6%)	116 (43.6%)	44 (45.4%)	906 (33.8%)	*p < 0.001*
		
	*No*	9 (47.4%)	150 (56.4%)	53 (54.6%)	1777 (66.2%)	

**Radiotherapy (N = 3058)**	*Yes*	6 (31.6%)	99 (37.1%)	43 (44.3%)	1203 (45.0%)	*0.061*
		
	*No*	13 (68.4%)	168 (62.9%)	54 (55.7%)	1472 (55.0%)	

* NES not elsewhere specified; NOS not otherwise specified.

In multivariate analyses adjusting for patient factors, disease factors and treatment, there was an overall significant difference among ethnic groups. For individual ethnic groups, only Chinese had significantly longer survival than the Other ethnicities, as shown in Table 2. This survival advantage in Chinese was only seen for non-metastatic disease (HR = 0.78, 95% CI; 0.64-0.95).

**Table 2:** Effect size of ethnicity for overall survival of gastric cancer patients

Ethnicity	N	HR	95% CI		p
**Iranian**	16	0.64	0.34	1.18	*P = 0.006*
	
**Chinese**	214	0.76	0.65	0.90	
	
**South Asian**	72	0.88	0.68	1.14	
	
**Other**	2038	Reference			

Hazard ratio (HR) and 95% confidence interval (CI) from Cox proportional hazards regression analysis adjusted for patient sex, patient age, year of diagnosis, tumor histology (Lauren), tumor location, tumor stage and treatment.

### Esophageal cancer

2873 cases of esophageal cancer were diagnosed during the study period. Descriptive characteristics of these patients are presented by ethnicity in Table 3. The majority of South Asians were women whereas the majority in the other ethnic groups were men (p < 0.01). There was no significant difference in age at diagnosis among the ethnic groups, the average age being 73.0 years, 68.0 years, 65.5 years and 68.4 years for Iranians, Chinese, South Asians and Other ethnicities, respectively. There was no significant difference among ethnic groups based on date of diagnosis.

**Table 3:** Descriptive characteristics for esophageal cancer by ethnicity

		Iranian	Chinese	South Asian	Other	P
**Sex (N = 2873)**	*Male*	10 (71.4%)	94 (74.6%)	57 (47.9%)	1821 (69.7%)	*p < 0.001*
		
	*Female*	4 (28.6%)	32 (25.4%)	62 (52.1%)	793 (30.3%)	

**Age in years (N = 2873)**	*Less than 55*	0 (0.0%)	14 (11.1%)	21 (17.6%)	314 (12.0%)	0.12
		
	*55-64*	1 (7.1%)	35 (27.8%)	32 (26.9%)	610 (23.3%)	
		
	*65-74*	9 (64.3%)	41 (32.5%)	35 (29.4%)	858 (32.8%)	
		
	*75 and More*	4 (28.6%)	36 (28.6%)	31 (26.1%)	832 (31.8%)	

**Years of Diagnosis (N = 2873)**	*1984-1990*	3 (21.4%)	16 (12.7%)	22 (18.5%)	486 (18.6%)	0.164
		
	*1991-1995*	1 (7.1%)	26 (20.6%)	15 (12.6%)	580 (22.2%)	
		
	*1996-2000*	3 (21.4%)	38 (30.2%)	33 (27.7%)	637 (24.4%)	
		
	*2001-2006*	7 (50%)	46 (36.5%)	49 (41.2%)	911 (34.9%)	

**Tumor Histology (N = 2873)**	SCC *	5 (35.7%)	103 (81.7%)	81 (68.1%)	1389 (53.1%)	*p < 0.001*
		
	AC **	7 (50.0%)	19 (15.1%)	27 (22.7%)	1101 (42.1%)	
		
	*Other*	2 (14.3%)	4 (3.2%)	11 (9.2%)	124 (4.8%)	

**Tumor Location (N = 2873)**	*Upper 1/3*	2 (14.3%)	23 (18.3%)	17 (14.3%)	314 (12.0%)	*p < 0.001*
		
	*Middle 1/3*	1 (7.1%)	45 (35.7%)	34 (28.6%)	605 (23.1%)	
		
	*Lower 1/3*	9 (64.3%)	40 (31.7%)	51 (42.9%)	1383 (52.9%)	
		
	NES/NOS***	2 (14.3%)	18 (14.3%)	17 (14.3%)	312 (12.0%)	

**Tumor Stage (N = 2594)**	*I*	1 (8.3%)	12 (10.3%)	8 (7.6%)	212 (9.0%)	0.84
		
	*II*	6 (50.0%)	66 (56.9%)	56 (53.3%)	1363 (57.8%)	
		
	*III*	3 (25.0%)	27 (23.3%)	26 (24.8%)	459 (19.4%)	
		
	*IV*	2 (16.7%)	11 (9.5%)	15 (14.3%)	326 (13.8%)	

**Surgery (N = 2830)**	*Yes*	2 (15.4%)	24 (19.2%)	35 (29.9%)	630 (24.5%)	0.23
		
	*No*	11 (84.6%)	101 (80.8%)	82 (70.1%)	1944 (75.5%)	

**Chemotherapy (N = 2820)**	*Yes*	0 (0.0%)	39 (31.2%)	25 (21.6%)	526 (20.5%)	*0.0084*
		
	*No*	13 (100.0%)	86 (68.8%)	91 (78.4%)	2039 (79.5%)	

**Radiotherapy (N = 2853)**	*Yes*	13 (100.0%)	112 (89.6%)	111 (93.3%)	2240 (86.3%)	0.052
		
	*No*	0 (0.0%)	13 (10.4%)	8 (6.7%)	355 (13.7%)	

*SCC squamous cell carcinoma

**AC adenocarcinoma

*** NES not elsewhere specified; NOS not otherwise specified

Tumour location was significantly different among ethnic groups (p < 0.01). More than half of tumors in Iranians and Other ethnicities were located lower third of the esophagus whereas this location was less common in Chinese and South Asians. Histology was significantly different among the ethnic groups (p < 0.01), with Chinese and South Asians having higher proportions of squamous cell carcinoma compared to Iranians and Other ethnicities.

**Figure 4:**
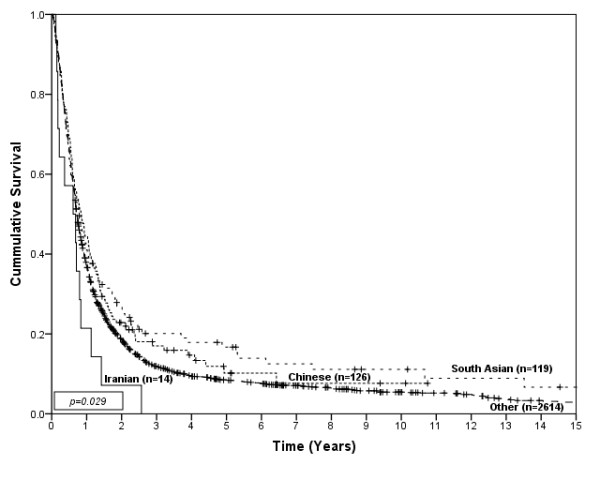
**Survival of esophageal cancer patients by ethnic group**.

**Figure 5:**
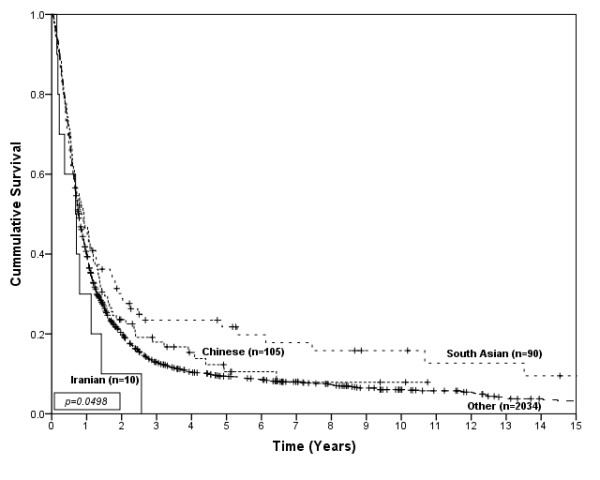
**Survival of esophageal cancer patients by ethnic group for non-metastatic disease**.

There were no significant differences in stage or the proportion with metastatic disease among ethnic groups. Treatment received was not different, except for chemotherapy which had significant differences among the ethnic groups (p < 0.01), with the Chinese, Iranian and South Asian patients accessing chemotherapy more often than Other ethnicities. 15% of Iranians, 45% of Chinese, 46% of South Asians and 42% of people in the Other category received more than one type of primary treatment. Iranians had median survival of 7 months (95%CI; 2.1-11.9), Chinese had a median survival of 10 months (95%CI; 7.0-12.9), South Asians had a median survival of 9 months (95%CI; 6.9-11.1) and people in the Other category had a median survival of 8 months (95%CI; 7.6-10.6). Figure 4 shows the survival curves for esophageal cancer patients by ethnic group (p = 0.029). In univariate analysis only South Asian (HR = 0.82, 95% CI; 0.67-1.00) showed slightly better survival comparing to Other. In multivariate analyses, South Asians showed better survival compared to the Other ethnicity group in the individual group comparison, however the overall difference among ethnic groups was not significant (Table 4). A significant survival difference only was observed among ethnic groups for patients with non-metastatic disease (p = 0.0498), as shown in Figure 5. Again, South Asians showed better survival compared to the Other ethnicity group (HR = 0.74, 95%CI; 0.56-0.97) in the multivariate analysis.

**Table 4:** Effect size of ethnicity for overall survival of esophageal cancer patients

Ethnicity	N	HR	95% CI		P
**Iranian**	10	1.13	0.61	2.12	
	
**Chinese**	95	0.9	0.72	1.13	0.14
	
**South Asian**	81	0.8	0.59	0.98	
	
**Other**	1947	Reference			

Hazard ratio (HR) and 95% confidence interval (CI) from Cox proportional hazards regression analysis adjusted for patient sex, patient age, year of diagnosis, tumor histology, tumor location, tumor stage and treatment.

## Discussion

An earlier population-based study in BC reported overall five-year survival rates of 8.8% for esophageal cancer and 16.2% for gastric cancer [21]. The current study was conducted to examine the effect of ethnicity on survival. The selection of ethnic groups was based on the predominant ethnic groups in the BC population and availability of ethnicity information. Our results indicate that patient ethnicity is a prognostic factor for both gastric and esophageal cancer; however ethnicity is only an independent prognostic factor for gastric cancer patients.

Ethnicity may represent biological characteristics of patients. Genetic variation may be responsible for differences in tumor-host interactions, such as the micro-architecture of tumors [22] and the complex process of metastasis, both of which are influenced by host genetic polymorphisms [23]. Ethnicity may also determine lifestyle and environmental characteristics including cultural, socioeconomic, and religious practices. Such differences are expected to be less apparent with increasing generations after immigration. Additionally, migration itself is one of the determinants of health outcome, and the "healthy migrant effect" could explain some of the observed survival difference among ethnic groups [24]. The difference in patient survival is not likely to be due to healthcare disparities among minority groups, as all BC residents receive free healthcare through the BC Medical Services Plan (MSP). Interestingly, survival was found to be better in minority groups compared to the BC general population.

Prognostic factors can be classified into three broad groups: i) tumor-related, ii) host-related, and iii) environment-related (including healthcare, treatment and lifestyle) factors [25,26]. Among tumor-related prognostic factors, disease stage is the most important [26] and often strongly influences the treatment plan. There were no significant differences in the stage distributions among ethnic groups; however, survival differences among ethnic groups were only significant for non-metastatic (i.e., stage I-III) disease. After adjustment for other factors (such as stage), the prognostic effect of ethnicity was significant only for gastric cancer patients.

Location of tumor (i.e., tumor topography) is a potential determinant of cancer survival. Our observation indicates significant differences in tumor location among different ethnic groups. It has been shown previously in Western countries that gastric cardia tumors are associated with worse survival compared to distal gastric tumors [27-29]. In addition, for studies of esophageal cancer, the location of tumors also showed differences in survival. Tumors in the middle 1/3 of the esophagus show worse survival in Turkey and Ardabil (Iran) [30,31], but tumors in the lower 1/3 of the esophagus are reported to have worse survival in BC and the United States[21,32].

Among host-related prognostic factors, ethnic differences were found for sex and age in both gastric and esophageal cancer. Of environment-related factors, treatment is likely the most powerful determinant of survival. There were significant ethnic differences in the proportions of gastric cancer patients who received surgery and chemotherapy. The reason for treatment differences among ethnic groups is not clear in a system where all patients have equal access to cancer care, but the differences might be explained by disease factors, other patient characteristics or patient preferences.

The result for gastric cancer is consistent with several US studies in which all other ethnic groups had better survival compared to the non-Hispanic white population [33], and a Los Angeles study that showed that Asians with gastric adenocarcinoma had superior outcomes compared to other ethnic groups [34]. Our study also confirms the findings of an earlier study in BC that reported better survival outcomes for gastric cancer patients with Asian ethnicity compared to the general population [35]. Our findings are consistent with international population-based cancer survival data that indicate that the 5-year survival for gastric cancer in China is higher than in India [36]. A comparison between registries from Shanghai (China) and Madras (India) shows that the 5-year relative survival for gastric (20% versus 7.5%) and esophageal cancer (9.0% versus 6.9%) is better in Shanghai [37]. These survival rates for both cancers are also higher than those reported in Iran [38].

It has been suggested that lower quality care and disparities in treatment are major contributors to differences in survival between minority and non-minority populations [39]. BC residents have access to publicly-funded healthcare, and the BC Cancer Agency (BCCA) has developed province-wide treatment guidelines and protocols [40].

### Strengths and limitations

The main strength of this study is the availability of reliable population-based data with details on tumor histology and pathology, treatment, disease stage and survival outcomes. The main limitation of this study is the lack of self-reported ethnicity information, requiring the use of a proxy method (i.e., name lists) to assign ethnicity. The weakness of using name lists as proxy for ethnicity is greater for women, who may change their surnames after marriage. Women account for only 30% of gastric and esophageal cancer cases in BC[21], but the possibility of misclassification in this subset must be considered. Based on a Statistics Canada report, visible minorities in Canada are a relatively young group and only 29% are older than 45 years, compared with 41% in the general population that are older than 45 http://www40.statcan.ca/l01/cst01/demo50a-eng.htm. Gastric and esophageal cancer is diagnosed at a late age and the observed survival differences between ethnicities in this study might be due to age distributions.

## Conclusions

Our study investigated ethnicity as a prognostic factor for gastric and esophageal cancer patients. It has been shown that for gastric cancer, patient ethnicity is significant and Chinese patients experience better survival than people from the Other ethnicity (i.e., non-South-Asian, non-Chinese and non-Iranian) group. Despite the observed survival benefit for gastric cancer patients who are Iranian, the low number of patients in this ethnic group does not permit a meaningful interpretation. Our results also indicate that, for esophageal cancer, South Asians have better survival compared to the Other ethnicity group.

Gastric and esophageal cancers are deadly diseases that are often diagnosed at a stage when the treatment options are limited and less effective. Ethnicity may represent underlying genetic factors. Such factors could influence host-tumor interactions by altering tumor etiology and therefore its chance of spreading. Alternatively, genetic factors may determine response to treatments. Finally, ethnicity may represent non-genetic factors that affect survival. Differences in survival by ethnicity support the importance of ethnicity as a prognostic factor, and may provide clues for the future identification of genetic or lifestyle factors that underlie these observations.

## List of abbreviations

BCCA: BC Cancer Agency; BCCR: BC Cancer Registry; CI: confidence interval; GI: gastrointestinal; HR: hazard ratio; ICD-O: International Classification of Diseases for Oncology; MSP: BC Medical Services Plan; NES: not elsewhere specified; NOS: not otherwise specified; SMPBC: Screening Mammography Program of British Columbia;

## Competing interests

The authors declare that they have no competing interests.

## Authors' contributions

MB designed the study, performed the analysis and wrote 100% of the manuscript. AS supervised clinical aspects of the study and reviewed the manuscript. GH supervised epidemiological aspects of the study and reviewed the manuscript. NL supervised statistical aspects of study and reviewed the manuscript. CB and ABW supervised all aspects of study, contributed to the interpretation of findings, and reviewed the manuscript.

## Pre-publication history

The pre-publication history for this paper can be accessed here:

http://www.biomedcentral.com/1471-2407/11/164/prepub
